# Chemokine CCL27 is a novel plasma biomarker for identification the nasopharyngeal carcinoma patients from the Epstein-Barr virus capsid antigen-specific IgA seropositive population

**DOI:** 10.1186/s12885-017-3718-2

**Published:** 2018-01-02

**Authors:** Min-jie Mao, Ning Xue, Xue-ping Wang, Pei-dong Chi, Yi-jun Liu, Qi Huang, Shu-qin Dai, Wan-li Liu

**Affiliations:** 1 Department of Laboratory Medicine, State Key Laboratory of Oncology in South China, Collaborative Innovation Center for Cancer Medicine, Sun Yat-sen University Cancer Center, Guangzhou, 510060 China; 2 Department of Laboratory Medicine, Affiliated Tumor Hospital of Zhengzhou University, Henan Tumor Hospital, Zhengzhou, 450100 China; 3 Guangdong Medical University, Guangzhou, 523808 China

**Keywords:** CCL27, VCA-IgA, Nasopharyngeal carcinoma, Early screening

## Abstract

**Background:**

To investigate the predictive value of chemokine CCL27 for identifying early stage nasopharyngeal carcinoma (NPC) patients within a population seropositive for Epstein-Barr virus (EBV) capsid antigen-specific IgA (VCA-IgA).

**Methods:**

CCL27 in plasma samples from 104 NPC patients, 112 VCA-IgA–positive healthy donors, and 140 VCA-IgA–negative normal subjects was measured by ELISA. Expression of CCL27 in nasopharyngeal tissue from 20 VCA-IgA–positive healthy donors and 20 NPC patients was examined by immunohistochemical staining.

**Results:**

Levels of CCL27 in the plasma of VCA-IgA–positive healthy donors (607.33 ± 218.81 pg/ml) were significantly higher than the levels in all NPC patients (437.09 ± 217.74, P = < 0.0001) and in the subset of patients with early stage NPC (463.85 ± 226.17, *P* = 0.0126). Plasma CCL27 levels were significantly lower in the VCA-IgA–negative normal subjects (358.22 ± 133.15 pg/ml) than in either the VCA-IgA–positive healthy donors (*P* < 0.0001) or the NPC patients (*P* = 0.0113). CCL27 protein was detected in 16 of 20 (80%) nasopharyngeal tissue samples from VCA-IgA–positive healthy donors and in 3 of 20 (15%) tumor tissue samples from NPC patients. There was no relationship between CCL27 levels and VCA-IgA titers or plasma EBV DNA content. Receiver operating characteristic (ROC) curves demonstrated that plasma CCL27 levels had a sensitivity of 67.00%, a specificity of 73.10%, and an area under the ROC of 0.725 (95% confidence interval [CI]: 0.657–0.793) for distinguishing between NPC patients and VCA-IgA–positive healthy donors. Further analysis showed that CCL27 levels could distinguish between early stage NPC patients and VCA-IgA–positive healthy donors with an area under the ROC of 0.712 (95% CI: 0.560–0.865), a sensitivity of 59.80%, and a specificity of 84.60%.

**Conclusions:**

Chemokine CCL27 could successfully identify NPC patients within a VCA-IgA–positive population.

## Background

Nasopharyngeal carcinoma (NPC) is one of the most common malignant neoplasms in South China and Southeast Asia. The etiology of NPC includes Epstein-Barr virus (EBV) infection, environmental and genetic factors, and dietary habits [1, 2]. Because EBV infection is closely associated with the occurrence of NPC, EBV-related biomarkers, such as EBV viral capsid antigen-specific IgA (VCA-IgA), have been widely used in NPC screening.

VCA-IgA shows good sensitivity but the false-positive rate in primary screening is high and the specificity for identification of NPC within EBV antibody-positive diseases is poor [3] (Lin et al., 1977) [4] reported that VCA-IgA has been measured during NPC screening of 413,164 subjects, of whom 12,629 cases were VCA-IgA positive (positive rate of 3.06%). After the last follow-up, only 174 of the 12,629 subjects were confirmed to have NPC (positive predictive value of 1.4%). In large population screens in Taiwan [5] and Hong Kong [6], the VCA-IgA–positive rate was 3–6% and the NPC-positive rate was 1.5–4.4% [7]. In our previous study, we used nasopharyngoscopy and serology to screen 28,688 individuals in Guangdong Sihui and Zhongshan from 2008 to 2010. Of the 3046 subjects who were VCA-IgA and/or EBNA1-IgA positive, NPC was diagnosed in 10.6%. After 6 years of follow-up, only 41 cases of NPC were verified, to give a positive predictive rate of only 1.3%. Thus, VCA-IgA and EBNA1-IgA can be used effectively to screen for NPC, but the positive predictive value for NPC is low [8]. Therefore, it is crucial to find new biomarkers that can identify NPC patients in the VCA-IgA–positive population.

Chemokines are small structurally related multifunctional proteins that play important roles in T cell development, differentiation, maturation, and trafficking, and other aspects of immune function [9]. Recent studies have shown that chemokines play an important role in the regulation of tumor progression, including proliferation and metastasis [10, 11]. CCL27 is a C-C motif chemokine also known as cutaneous T cell-attracting chemokine (CTACK). The only known receptor for CCL27 is CCR10, which is expressed in normal skin, which can make a small amount of CCR10+/CLA+ memory T cells homing to the inflammation microenvironment to maintain immune surveillance. However, the predictive value of CCL27 in NPC remains unclear [12, 13].

To shed light on the association between CCL27 and NPC, we measure plasma CCL27 levels in NPC patients, VCA-IgA–positive healthy donors, and VCA-IgA–negative normal subjects, and evaluated the diagnostic performance of CCL27 for NPC detection in a VCA-IgA–positive population.

## Methods

### Patients

Between September 2015 and December 2015, 104 NPC patients (median age 45 years, range 23–69 years; 79 men and 25 women) at the Sun Yat-Sen University Cancer Center were enrolled in this study. The inclusion criteria for NPC were as follows: all of the patients met the diagnostic criteria for NPC (TNM staging defined by the 2009 Union for International Cancer Control/American Joint Committee on Cancer staging system for NPC), and all 104 NPC patients were seropositive for EBV VCA-IgA. Exclusion criteria were as follows: (1) patients who received chemoradiotherapy or surgery before enrolling in this study; (2) patients who with concomitant diseases, such as skin disease or another type of malignancy. We also enrolled 112 VCA-IgA–positive cancer-free healthy donors (median age 41 years, range 27–65 years, 64 men and 48 women), who were followed up for 6 months to exclude cancer and inflammation-related diseases. A VCA-IgA–negative control population consisted of 140 normal subjects who were free of detectable infection, cancer, or other known disease. Samples from the healthy donors and normal subjects were collected from the physical examination department at the Sun Yat-Sen University Cancer Center. Venous blood samples (2–4 ml) obtained from the NPC patients at the time of diagnosis (before treatment) were mixed with EDTA-K_2_ anticoagulant, centrifuged at 3000 rpm for 10 min, and stored at −80 °C until use. Written informed consent for the use of plasma and tissue samples was obtained from all patients and healthy participants. This study was approved by the Institute Research Ethics Committee of the Sun Yat-Sen University Cancer Center, Guangzhou, China.

### Tissue specimens

For immunochemistry, formalin-fixed and paraffin-embedded nasopharyngeal tissue from 20 NPC patients and 20 VCA-IgA–positive healthy donors were obtained from the Sun Yat-Sen University Cancer Center. All NPC samples were collected immediately after surgical resection and the diagnosis were confirmed by pathological review.

### ELISA assay

Plasma CCL27 concentrations were measured using a double-antibody sandwich ELISA according to the manufacturer’s instructions (R&D Systems, Minneapolis, MN, USA). In brief, 100 μl/well of the capture antibody (mouse anti-human CCL27, 4.0 μg/ml) was added to 96-well microplates overnight at room temperature. Test samples or CCL27 standard (100 μl/well) were added to the wells and the plates were incubated for 2 h. Detection antibody (biotinylated goat anti-human CCL27, 75 ng/ml) was added at 100 μl/well and the plate was incubated for 2 h. Finally, 100 μl/well of horseradish peroxidase-conjugated streptavidin was added to each well. After addition of a colorimetric reagent for 0.5 h, the reaction was stopped by the addition of 2 N sulfuric acid and the absorbance was measured at 450 nm. Each test included a standard control (coefficient of variation = 12%).

### Immunohistochemical staining

Nasopharyngeal tissue sections were incubated with a goat anti-CCL27 antibody (1:20, R&D Systems) overnight at 4 °C. The samples were washed and the chromogenic reaction step was performed using a PV-9001 Polymer Detection System kit for immunohistochemical staining (Beijing Golden Bridge Biotechnology, China).

### Statistical analysis

Statistical analysis was performed with SPSS 16.0 for Windows software (SPSS, Chicago, IL, USA). Relationships between CCL27 protein expression and clinicopathologic features were analyzed by the Mann–Whitney U test. Comparison of CCL27 concentrations and EBV DNA copies between groups was assessed using the Kruskal–Wallis test. The diagnostic value of CCL27 was assessed by area under the receiver operating characteristic (ROC) curve (AUC). The cut-off value for CCL27 discrimination was defined by maximization of the Youden index. *P* values <0.05 were considered statistically significant. All reported *P* values are two sided.

## Results

### Relationship between plasma CCL27 levels and patient clinicopathological characteristics

The associations between the median plasma CCL27 concentrations and clinical variables for the 104 NPC patients are presented in Table 1.

**Table 1:** Associations between plasma CCL27 levels and clinical characteristics of the patients with NPC

Characteristics	No.	Median(range)	P value
Age			0.8556
≤45	52	405.35(107.33-1296.94)	
>45	52	383.76(84.03-872.33)	
Sex			0.5555
Female	25	375.12(107.33-917.17)	
Male	79	403.27(84.03-1296.94)	
pT stage			0.8991
PT1-pT2	25	403.27(217.84-1067.05)	
pT3	47	403.96(84.03-1296.94)	
pT4	32	395.68(108.98-872.26)	
pN stage			0.1946
pN 0/1	53	373.05(84.03-1067.05)	
pN 2/3	51	420.32(109.05-1296.94)	
Stage			0.9685
I + II	13	376.25(227.77-1067.05)	
III	50	397.70(84.03-1296.94)	
IV	41	403.27(108.98-872.26)	

### Plasma levels of CCL27 in NPC patients, VCA-IgA–positive healthy donors, and VCA-IgA–negative normal subjects

**Fig. 1:**
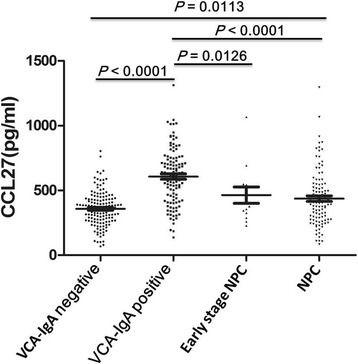
Plasma CCL27 levels in study subjects. VCA-IgA–negative normal subjects, *n* = 140; VCA-IgA–positive healthy donors, *n* = 112; early stage NPC patients, *n* = 13; and all NPC patients, *n* = 104

As shown in Fig. 1, the plasma levels of CCL27 in VCA-IgA–positive healthy donors (607.33 ± 218.81 pg/ml) were significantly higher than the pre-treatment levels in all NPC patients (437.09 ± 217.74 pg/ml, *P* < 0.0001) and the subset of patients with early stage NPC (Stage I + II, 463.85 ± 226.17, *P* = 0.0126). Furthermore, plasma CCL27 levels in VCA-IgA–negative normal subjects (358.22 ± 133.15 pg/ml) were significantly lower than the levels in either the VCA-IgA–positive healthy donors (*P* < 0.0001) or the NPC patients (*P* = 0.0113).

### Expression of CCL27 in nasopharyngeal epithelial tissue from NPC patients and VCA-IgA–positive healthy donors

**Fig. 2:**
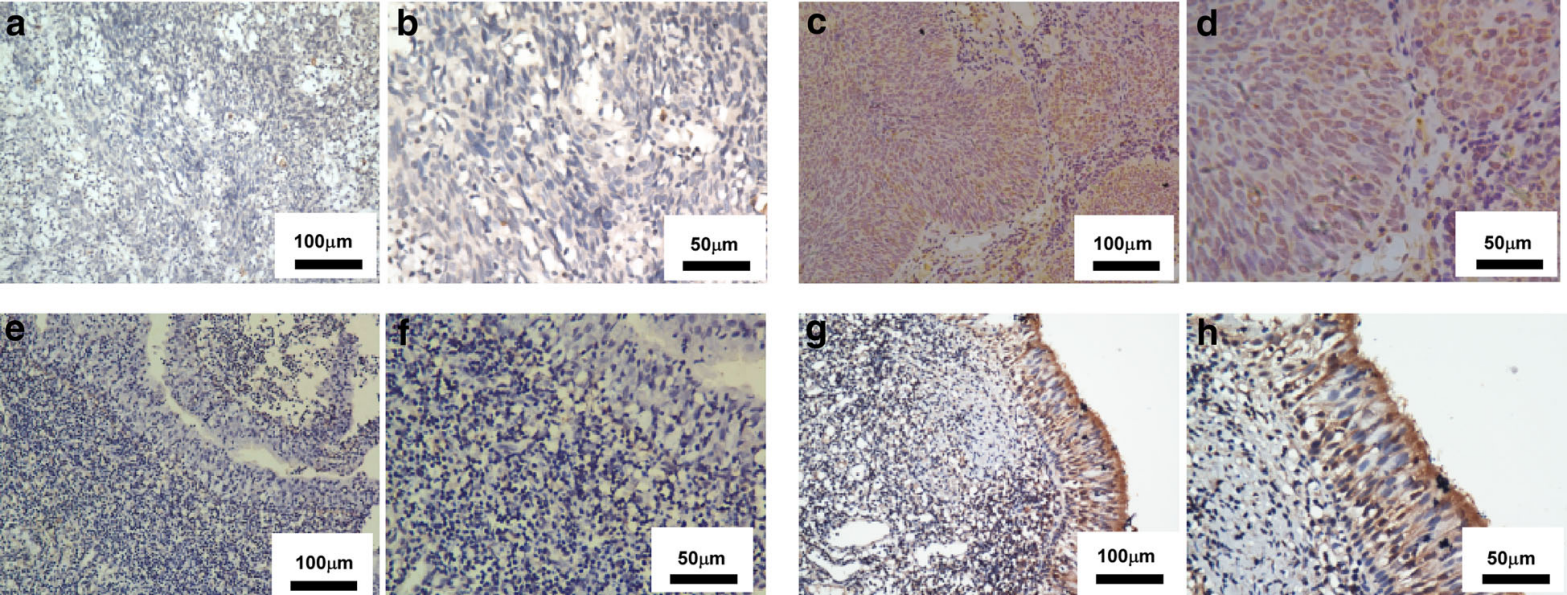
Expression of CCL27 in nasopharyngeal epithelial tissue from a NPC patient and a VCA-IgA–positive healthy donor. **a–h** Representative images showing immunohistochemical staining of CCL27 in NPC tumor tissue (low CCL27 expression: a-b, high CCL27 expression:c-d) and VCA-IgA–positive healthy tissue (low CCL27 expression: e-f, high CCL27 expression: g-h). Scale bars: a and c = 100 μm; b and d = 50 μm

We investigated the expression of CCL27 protein in nasopharyngeal tissue from healthy donors and NPC patients by immunohistochemical staining. High expression of CCL27 protein was observed in 16 of 20 (80%) in nasopharyngeal epithelium from VCA-IgA–positive healthy donors, and no expression of CCL27 protein was observed in 17 of 20 (85%) tumor tissues from NPC patients. CCL27 was located mainly in the cytoplasm of the nasopharyngeal epithelial cells (Fig 2).

### Relationship between CCL27 level, VCA-IgA titer, and EBV DNA content

**Fig. 3:**
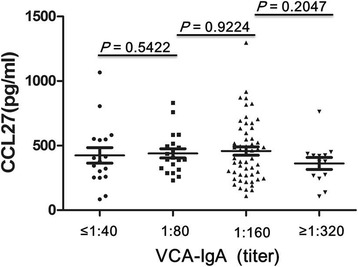
The relationship between plasma CCL27 levels and VCA-IgA titers in NPC patients. Patients were assigned to four groups based on VCA-IgA titers: ≤1:40, 1:80, 1:160, and ≥1:320

**Fig. 4:**
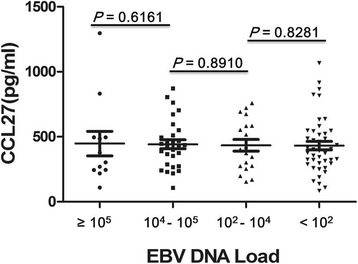
The relationship between plasma CCL27 levels and plasma EBV DNA content in NPC patients. Patients were assigned to four groups based on EBV DNA load: ≥10^5^ (*n* = 11), 10^4^–10^5^ (*n* = 26), 10^2^–10^4^ (*n* = 16), and <10^2^ (*n* = 36)

To assess the relationship between CCL27 levels and VCA-IgA titer, the NPC patients were assigned to four groups based on VCA-IgA titers: ≤1:40, 1:80, 1:160, and ≥1:320. There were no significant differences in plasma CCL27 levels among the four groups (*P* > 0.05), suggesting that CCL27 levels are unlikely to be directly related to the VCA-IgA titer (Fig 3). Similar results were obtained when the NPC patients were assigned to four groups based on plasma EBV DNA copy number: ≥10^5^ (*n* = 11), 10^4^–10^5^ (*n* = 26), 10^2^–10^4^ (*n* = 16), and <10^2^ (*n* = 36). Here, too, we observed no significant differences in plasma CCL27 levels among the four groups (*P* > 0.05), suggesting that CCL27 levels are unlikely to be directly related to EBV DNA copy numbers (Fig 4).

### Diagnostic performance of plasma CCL27 levels in identifying NPC patients in a VCA-IgA–positive population

**Fig. 5:**
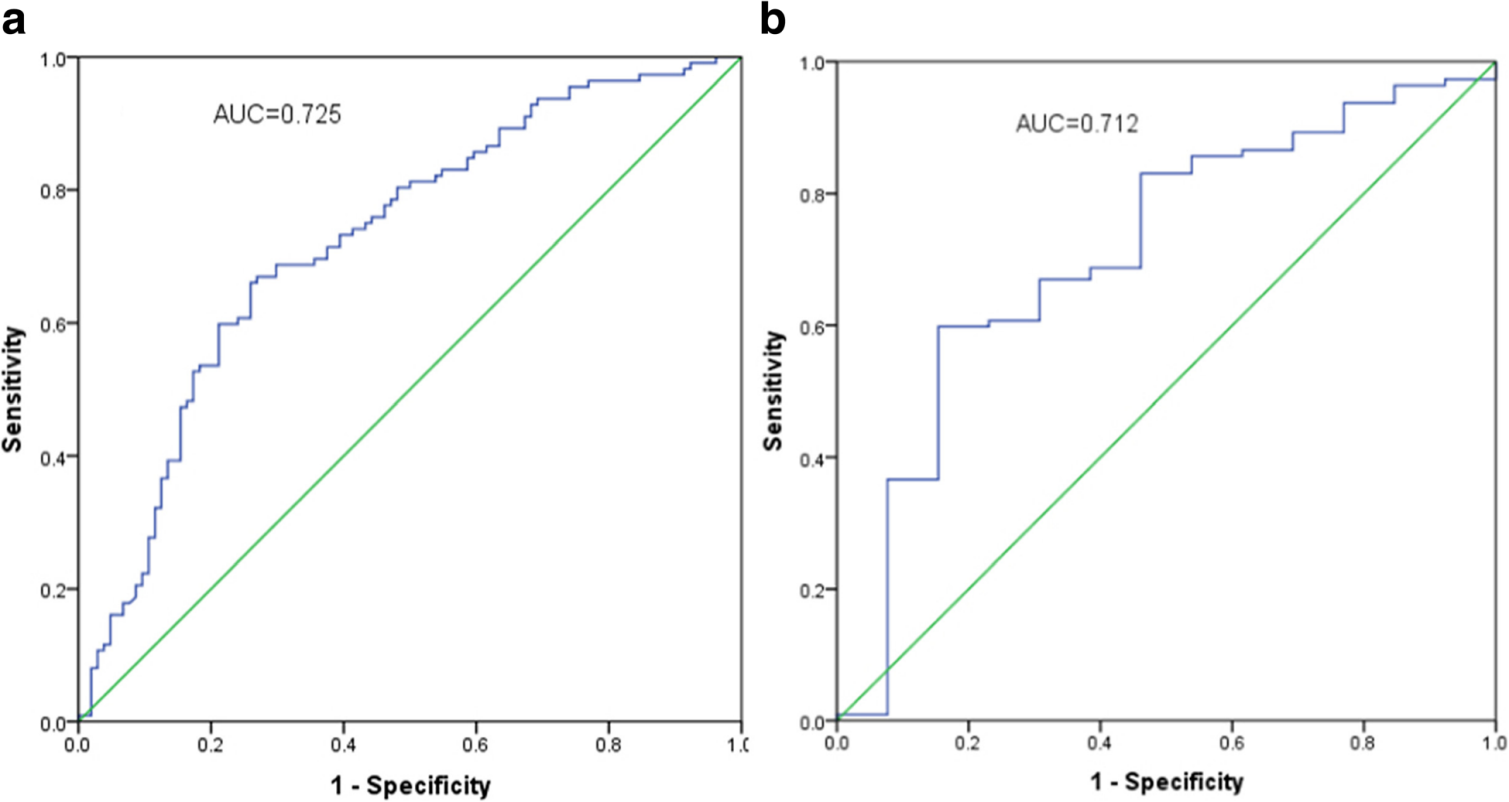
Diagnostic performance of plasma CCL27 levels. (a and b) ROC curves showing the diagnostic value of plasma CCL27 levels for distinguishing between VCA-IgA–positive healthy donors and (**a**) all NPC patients or (**b**) early stage NPC patients

ROC curves were plotted to identify a cut-off value that could distinguish the NPC patients from the VCA-IgA–positive healthy donors. As shown in Fig. 5a, the optimal cut-off value was 516.98 pg/ml CCL27 (AUC = 0.725, 95% CI: 0.657–0.793), with a sensitivity of 67.00% and a specificity of 73.10%. Further analysis showed that CCL27 levels could also distinguish between the early stage NPC patients and the VCA-IgA–positive healthy donors, with a cut-off value of 552.71 pg/ml (AUC = 0.712, 95% CI: 0.560–0.865), a sensitivity of 59.80%, and a specificity of 84.60% (Fig 5b).

## Discussion

NPC is a nasopharyngeal epithelial cell malignancy. In southern China, it is the most common form of head and neck cancer. The incidence of the disease is closely associated with infection with EBV, a herpes virus [14]. After infection, viral products can influence cell proliferation, apoptosis and gene mutation, which might contribute to malignant transformation [15]. EBV DNA copy numbers and VCA-IgA antibody titers can indirectly reflect the activation state of EBV in the body [16–18]. CCL27 is a C-C motif chemokine, and the rationales to pick up CCL27 are as follows: Firstly, we have done gene chip detection between 8 NPC patients and 8 VCA-positive healthy donors, and found that CCL27 is down regulated in NPC patients; Secondly, we have tried our best to consult the reference: CCL27 is increased in inflammatory skin diseases, such as psoriasis, atopic dermatitis, and contact dermatitis, as well as other types of skin inflammation associated with T lymphocyte infiltration. Classic CCL27 contains secreting peptide,which has chemotaxis to the activation of CD4 + T cells, and plays a major role on skin inflammation, mainly produced by the skin keratinocyte cell. CCL27 has also been reported to be highly expressed in squamous cell epithelial cells and melanoma cells, and NPC is non- squamous type; in the latter, it may be involved in invasion and metastasis [19].

This study is the first to investigate CCL27 as a potential biomarker for use in primary screening for NPC, especially in VCA-IgA–positive individuals. The results show that CCL27 levels were significantly higher in EBV-infected individuals (i.e., NPC patients and VCA-IgA–positive healthy donors) than in uninfected normal subjects. The main mechanisms were that CCL27 will be increased according to the high expressed of CCR10, which is upregulated on T cells immortalized by EBV infection. We also found that plasma CCL27 concentrations were higher in the VCA-IgA–positive healthy donors than in the NPC patients, for which there are several potential explanations. First, EBV infection induces an immune response that, in a normal situation, would increase CCL27 levels for recruitment of T cells [20]; however, CCL27 concentrations may be lower in subjects with abnormal immune function [21]. Second, Pivarcsi et al. [22] reported that reduced levels of cytokines and chemokines, such as CCL27, could allow tumors to evade the immune system. Compared with normal skin, keratinocyte-derived cutaneous tumor cells may downregulate the expression of CCL27 via the epidermal growth factor receptor–Ras–MARK signaling pathway, thereby evading the T cell-dependent antitumor immune response [23].

In recent years, several methods have been routinely used to diagnose NPC, such as nasopharyngoscopy, imaging modalities, anti-EBV antibody detection, and EBV DNA quantification [24]. However, these methods have limited sensitivity and specificity and are not entirely reliable, and the gold standard for diagnosis is nasopharyngeal lesion biopsy. VCA, the capsid antigen, and EA, the early antigen, are released soon after EBV infection. VCA is strongly immunogenic, more than 90% of NPC patients are VCA-IgA positive, and the levels can be reduced by treatment. Therefore, VCA-IgA could function as an NPC screening marker, an independent prognostic factor, and a predictive index in NPC therapy. EA-specific IgA, which is produced when EBV begins to replicate, has high specificity but limited sensitivity for the detection of early NPC and it is mismatched with the severity of NPC [25]. EBV DNA is integrated into the lymphocyte genome and is also present in the cytoplasm in the form of circular DNA. Thus, EBV DNA copy number in the plasma is a direct reflection of the EBV load in the patient [26, 27]. However, Han et al. [1] systematically reviewed 18 studies from China and elsewhere and found a positive predictive value of VCA-IgA for NPC of only 8.84%. Collectively, the combination of the poor specificity of VCA-IgA, the limited sensitivity of EA-IgA, the low diagnostic rate of EBV DNA content, and invasion of pathological biopsies means that there is no suitable method for screening of the general population for NPC. Therefore, it is critical to find a biomarker able to identify NPC patients in the VCA-IgA–positive population.

In our study, ROC curves showed that plasma CCL27 concentrations could effectively differentiate NPC patients from the VCA-IgA–positive healthy donors (AUC = 0.725, 95% CI: 0.657–0.793) with a sensitivity of 67.00% and a specificity of 73.10%. Moreover, CCL27 could also distinguish between early stage NPC patients and the VCA-IgA–positive healthy donors (AUC = 0.712, 95% CI: 0.560–0.865) with a sensitivity of 59.80% and a specificity of 84.60%. These results indicate that CCL27 could be used as a biomarker to identify NPC patients, and served as the complement of *VCA-IgA titers.*

However, there were no significant correlations between plasma CCL27 levels and either VCA-IgA titer or EBV DNA copy number. This may be because EBV VCA-IgA is produced in the early stage of infection and has a longer half-life than CCL27; indeed, VCA-IgA is present at high concentrations even after EBV is cleared from the body. CCL27 is involved in the general immune response and is a dominant player in tumor immunity; thus, its level might not be associated with a change in EBV VCA-IgA [28]. As a cellular marker, CCL27 differs from the traditional EBV markers. The combination of viral markers and cellular markers could provide a new and effective method to diagnose NPC, with CCL27 complementing the more traditional biomarkers such as VCA-IgA and EA-IgA. Detection of VCA-IgA and EA-IgA is complex, objective, which have less precision and accuracy. In contrast, detection of CCL27 in plasma can be achieved with good accuracy and reproducibility and it does not require specialized equipment. Thus, it could be a convenient monitoring biomarker in routine examinations.

## Conclusions

The major limitations of this study are as follows: First, Our study is a single-center analysis in our hospital, and need to be validated in large prospective trials of multicenter; Second, our study is a prospective analysis, we plan to collect complete patient data pre- and post-treatment to further evaluate the prognostic value and biological function of CCL27 in NPC; Third, although our study found that CCL27 could be a useful biomarker for identifying NPC patients within a VCA-IgA–positive population, but the mechanisms of how CCL27 influences NPC patients is not clear, further studies aim at investigating the role of CCL27 in NPC. Our study is the first to investigate CCL27 as a potential complement biomarker for use in primary screening for NPC, especially in VCA-IgA–positive individuals, which was used to identify NPC and VCA-IgA–positive healthy donors and improve the positive predictive value of VCA-IgA in NPC.

## Abbreviations

CCL27: C-C Motif Chemokine Ligand 27
CTACK: Cutaneous T cell-attracting chemokine
EBV: Epstein-Barr virus
NPC: Nasopharyngeal carcinoma
VCA-IgA: Viral capsid antigen-specific IgA
